# Evaluation of Pyrrole Heterocyclic Derivatives as Selective MAO-B Inhibitors and Neuroprotectors

**DOI:** 10.3390/molecules31010186

**Published:** 2026-01-04

**Authors:** Maya Georgieva, Martin Sharkov, Emilio Mateev, Alexandrina Mateeva, Magdalena Kondeva-Burdina

**Affiliations:** 1 Department of Pharmaceutical Chemistry, Faculty of Pharmacy, Medical University of Sofia, 2 Dunav Str., 1000 Sofia, Bulgaria; 2 Department of Pharmacology, Pharmacotherapy and Toxicology, Faculty of Pharmacy, Medical University of Sofia, 2 Dunav Str., 1000 Sofia, Bulgaria

**Keywords:** pyrrole, MAO, neurotoxicity, neuroprotection, biotransformation

## Abstract

Novel pyrrole-based derivatives were synthesized in high purity and yields (52–89%), with **17i** and **17j** displaying selective MAO-B inhibition (50–60%), comparable to Selegiline, and negligible MAO-A activity. In rat brain subcellular fractions, both compounds showed low intrinsic neurotoxicity at 100 μM while exerting significant neuroprotective and antioxidant effects under 6-OHDA, t-BuOOH, and Fe^2+^/ascorbate-induced stress. Mechanistic studies indicate dual protection via reactive oxygen species scavenging and preservation of reduced glutathione, with mitochondria and microsomes being the most responsive compartments. The performed in silico analysis revealed no general toxicity alerts, though hydrazine groups classify the compounds as contact allergens, and the furan ring in **17i** poses hepatotoxic and carcinogenic risks. Metabolic predictions suggest ester hydrolysis at the pyrrole ring as the main biotransformation pathway. Overall, **17i** and **17j** are promising lead compounds for developing therapeutics targeting oxidative stress-related neurodegenerative diseases, such as Parkinson’s and Alzheimer’s, supporting further in vivo studies.

## 1. Introduction

Monoamine oxidases (MAOs) are mitochondrial outer membrane enzymes responsible for the oxidative deamination of neurotransmitters, such as serotonin, dopamine, and norepinephrine, producing hydrogen peroxide (H_2_O_2_) as a byproduct [1]. Two isoenzymes, MAO-A and MAO-B, were identified in 1968 and differ in substrate specificity and tissue distribution. MAO-A is expressed in neurons, astroglial cells, and peripheral tissues, including the gastrointestinal tract, liver, lung, and placenta, while MAO-B is present in the CNS and platelets [2]. Developmental studies show differential age-dependent expression. MAO-A is high at birth (78% of adult levels) and stabilizes in adulthood, whereas MAO-B is nearly absent at birth, rises in the first two years, and continues to increase ~20% per decade after age 18 [3]. Given their central role in neurotransmitter catabolism, MAOs are key pharmacological targets in neurological disorders.

Aberrant MAO activity contributes to neurodegeneration by increasing toxic aldehydes and H_2_O_2_, promoting oxidative stress, a critical factor in neuronal damage [4]. Overexpression of MAO-B in astrocytes, particularly in the hippocampus and cortex of Alzheimer’s disease (AD) brains, accelerates free radical generation and disrupts monoamine metabolism. MAO-A inhibitors primarily modulate serotonin and are used in depression, whereas MAO-B inhibitors target dopamine, making them relevant in Parkinson’s disease (PD). Non-selective MAO inhibitors affect multiple monoamines, offering broader therapeutic potential [5].

MAO inhibitors (MAOIs) were among the first antidepressants introduced in the 1950s and remain clinically relevant for atypical depression, panic disorder, social anxiety, and treatment-resistant depression [6,7]. Selective MAO-B inhibitors, such as Selegiline and Rasagiline, show symptomatic benefit in early-stage PD, reducing motor deficits and levodopa requirements without increasing morbidity or mortality [8]. Non-selective MAOIs (isocarboxazid, phenelzine, tranylcypromine, nialamide) and MAO-A inhibitors (moclobemide, toloxatone) are mainly used in mood disorders, whereas MAO-B inhibitors are essential in managing neurodegenerative conditions.

Heterocyclic compounds are key scaffolds in medicinal chemistry due to their structural diversity, stability, and versatility as pharmacophores. Nitrogen-containing heterocycles are widespread in natural products, drugs, vitamins, nucleic acids, and agrochemicals [9,10]. Synthetic heterocycles have demonstrated inhibitory effects on therapeutic enzymatic targets in AD and PD, highlighting their potential as neuroprotective agents [11]. Their structural diversity and adaptability make heterocycles attractive building blocks for novel therapies targeting neurodegenerative disorders [12,13,14].

This study focuses on the design and evaluation of newly synthesized pyrrole-based derivatives as potential neuroprotective agents, aiming to identify novel molecules suitable for the prevention and treatment of Alzheimer’s and Parkinson’s disease.

## 2. Results

### 2.1. Synthesis of the Target Derivatives

**Scheme 1:**
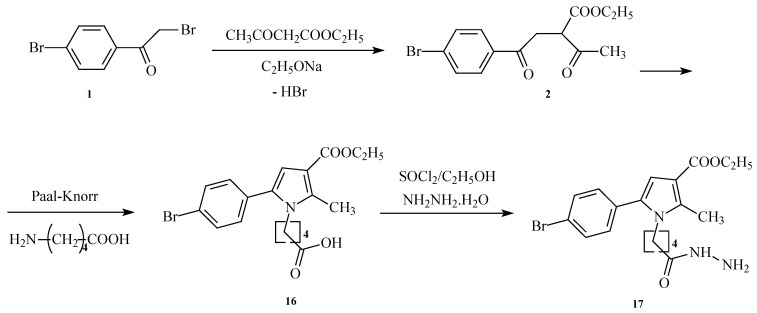
Synthesis of the target pyrrole heterocyclic hydrazide.

The synthesis of the target for the evaluations of pyrrole heterocyclic hydrazide was conducted as per the synthetic procedure presented in Scheme 1.

**Figure 1:**
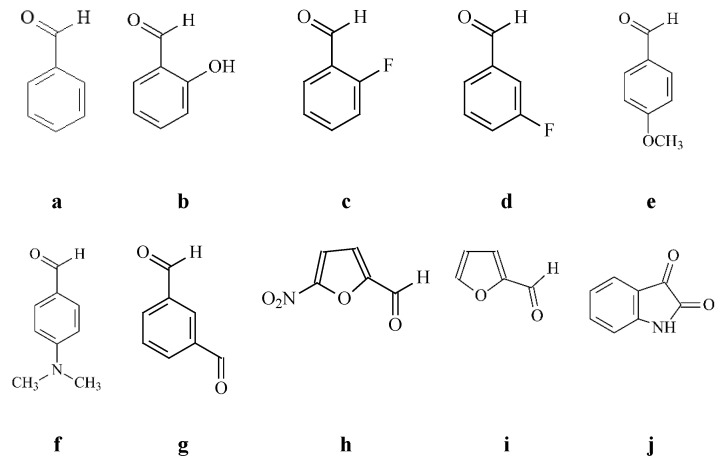
Structure of the used carbonyl partners (**a**–**j**).

The target hydrazones were obtained by a general procedure of condensation of the target hydrazine **17** in equimolar amounts with any of the carbonyl partners shown in Figure 1.

**Scheme 2:**
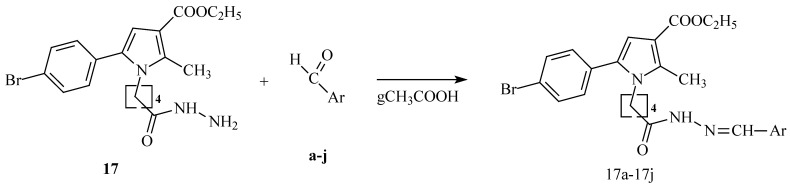
General scheme of synthesis of the target pyrrole-based hydrazones **17a**−**17j**.

The planned hydrazones were obtained as per the procedure demonstrated in Scheme 2.

The target final derivatives, together with the necessary intermediates, were chemically characterized through corresponding spectral methods, including IR, ^1^H, and ^13^CNMR spectral data. The corresponding ^1^H and ^13^CNMR spectra for the most active compounds are present in the Supplementary File (Supplementary Figures S1–S4, respectively). The purity of the compounds was elucidated and proven by their melting points, TLC characteristics (R_f_), and MS data. The corresponding yields for the purified products were also defined. All characterization data are presented in Section 4 and in Table 1, respectively.

### 2.2. Pharmacological In Vitro Assessment of the Target Pyrrole-Based Derivatives

#### 2.2.1. In Vitro Evaluation of MAO Inhibitory Activity

**Figure 2:**
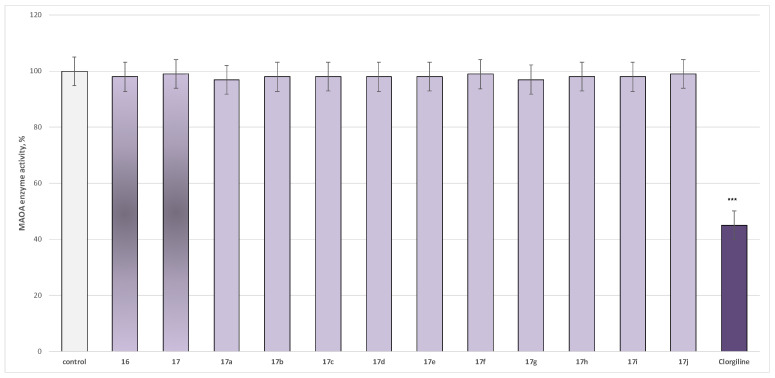
Effects of the synthesized compounds and Clorgiline (applied alone at a concentration of 1 μM) on *h*MAO-A activity. *** *p* < 0.001 against control (pure *h*MAO-A).

**Figure 3:**
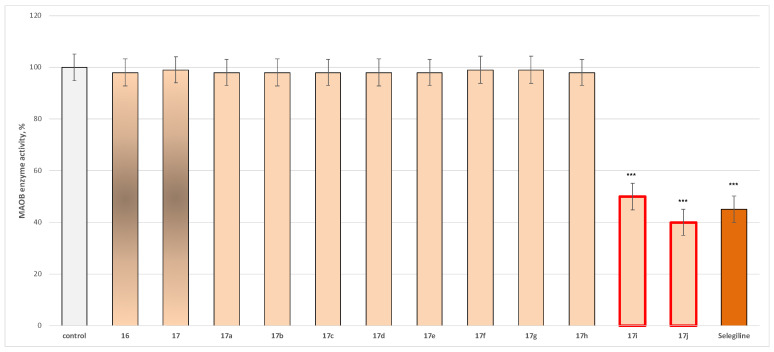
Effects of the synthesized compounds and Selegiline (applied alone at a concentration of 1 μM) on *h*MAO-B activity. *** *p* < 0.001 against control (pure *h*MAO-B).

In the current paper, we evaluated the effect of the target azomethine derivatives, together with the initial hydrazide, on both isoforms. The results are presented in Figure 2 for MAO-A effects and in Figure 3 for MAO-B activity.

The newly obtained target compounds did not exhibit statistically significant MAO-A inhibitory activity, with only the classical MAO-A inhibitor Clorgiline reducing enzyme activity by 55% relative to the control (pure *h*MAO-A) (Figure 2).

Within the evaluated molecules, two compounds, **17i** and **17j**, exhibited statistically significant MAO-B inhibitory activity, with **17j** demonstrating superior MAO-B inhibitory activity compared to Selegiline (Figure 3).

In addition, the corresponding IC_50_ of the most active representatives was determined, and the results are presented in Table 2, together with the calculated selectivity index.

The target selectivity index was calculated using the expression presented below:

**SI**: *h*MAO-B selectivity index = IC_50_(*h*MAO-A)/IC_50_(*h*MAO-B) (1)

#### 2.2.2. Neurotoxicity Determination

- Neurotoxicity determination of isolated rat brain synaptosomes

**Figure 4:**
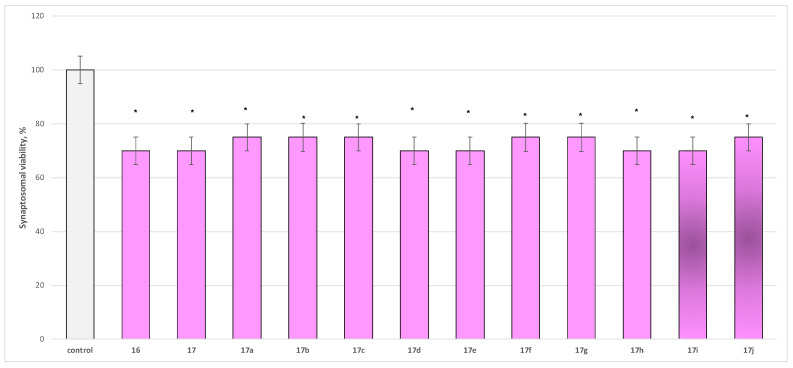
Effects of the synthesized compounds applied alone at a concentration of 100 μM on synaptosomal viability. * *p* < 0.05 against the control (untreated synaptosomes).

**Figure 5:**
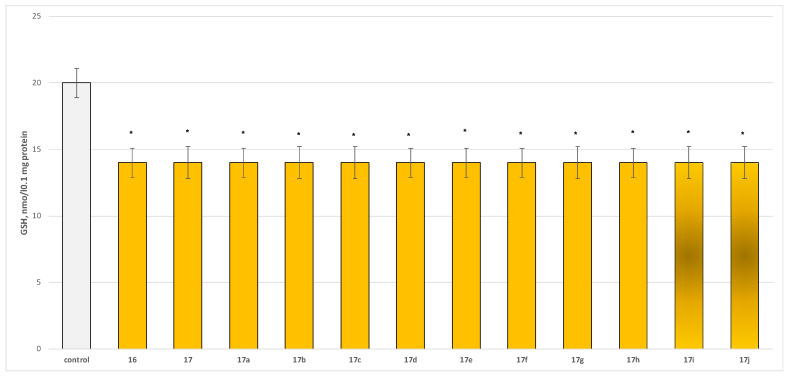
Effects of the synthesized compounds applied alone at a concentration of 100 μM on levels of reduced glutathione (GSH) in isolated rat brain synaptosomes. * *p* < 0.05 against the control (untreated synaptosomes).

To evaluate the safety profile of the synthesized derivatives **16**, **17,** and **17a**−**17j,** their independent effects on parameters characterizing the functional–metabolic profile of synaptosomes were assessed, specifically synaptosomal viability (Figure 4) and the level of reduced glutathione (GSH) (Figure 5).

The compounds exhibited low to moderate but statistically significant neurotoxicity relative to the control (untreated synaptosomes), reducing synaptosomal viability by 25–30% (Figure 4) and GSH levels by approximately 30% compared with the control (Figure 5).

- Neurotoxicity determination of isolated rat brain mitochondria

**Figure 6:**
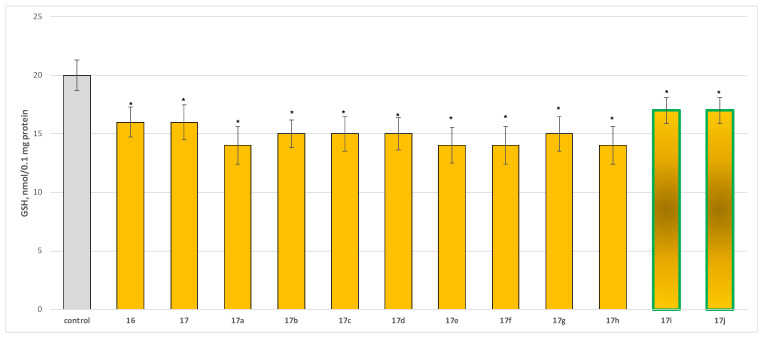
Effects of the synthesized compounds applied alone at a concentration of 100 μM on levels of reduced glutathione (GSH) in isolated rat brain mitochondria. * *p* < 0.05 against the control (untreated mitochondria).

**Figure 7:**
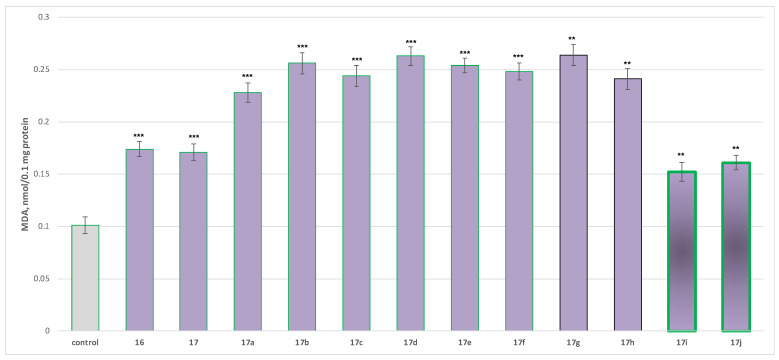
Effects of the synthesized compounds applied alone at a concentration of 100 μM on MDA production in isolated rat brain mitochondria. ** *p* < 0.01; *** *p* < 0.001 against the control (untreated mitochondria).

Two characteristic parameters were tested for assessment of the neurotoxic effects on isolated rat brain mitochondria—reduced GSH levels and changes in the MDA production (Figure 6 and Figure 7).

As the results indicate, tested compounds **17a**, **17e**, **17f**, and **17h** decrease the reduced glutathione (GSH) levels by ~30%; **17b**, **17c**, **17d**, and **17g** by ~25%; **17i** and **17j** by ~15%; and **17** and **16** by ~20% relative to untreated mitochondria (Figure 6).

Malondialdehyde (MDA) production was significantly elevated, ranging from 126 to 161% for the more toxic derivatives (**17a**–**17h**), 50 to 59% for **17i** and **17j**, and 69 to 72% for **17** and **16** compared to control mitochondria (Figure 7).

In summary, when administered alone at 100 µM, the obtained compounds produced a statistically significant but low to moderate neurotoxic effect on parameters reflecting the functional–metabolic status of isolated rat brain mitochondria. The least toxic were again **17i** and **17j**, with effects compared to the initial condensed pyrrole-based acid **16** (Figure 6 and Figure 7).

- Neurotoxicity determination of isolated rat brain microsomes

**Figure 8:**
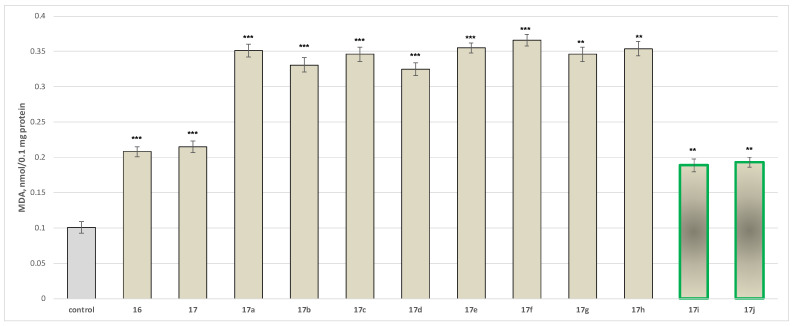
Effects of the synthesized compounds applied alone at a concentration of 100 μM on MDA production in isolated rat brain microsomes. ** *p* < 0.01; *** *p* < 0.001 against the control (untreated microsomes).

Applied individually at a concentration of 100 µM, the target compounds also demonstrated a statistically significant yet modest pro-oxidant effect on isolated rat brain microsomes. Among these, **17i** and **17j** exhibited the lowest cytotoxicity. All effects were evaluated in comparison with those of native N-pyrrolyl-carboxylic acid **16** (Figure 8).

The tested compounds **17a** increased MDA production by a statistically significant 248%; **17b** by 228%, **17c** by 243%, **17d** by 222%, **17e** by 251%, **17f** by 262%, **17g** by 243%, **17h** by 250%, **17i** by 87%, **17j** by 91%, **17** by 106%, and **16** by 113% relative to the control (untreated microsomes) (Figure 8).

The overall results indicate that when applied alone at a 100 μM concentration, the tested compounds exert statistically significant low to moderate neurotoxic and pro-oxidant effects on isolated rat brain microsomes. The analysis defined that among the tested series, the compounds exhibiting the lowest toxicity are **17i** and **17j**.

#### 2.2.3. Neuroprotective Properties

- Neuroprotection properties in isolated rat brain synaptosomes

For the evaluation of the protective ability of the target hydrazones, the in vitro 6-OHDA-induced neurotoxicity model was applied. The model mimics the neurodegenerative processes primarily observed in Parkinson’s disease (PD). The metabolism of 6-OHDA produces reactive quinones, which in turn generate reactive oxygen species (ROS). These reactive metabolites and ROS cause damage to both pre- and post-synaptic membranes, ultimately leading to neuronal cell damage [15].

**Figure 9:**
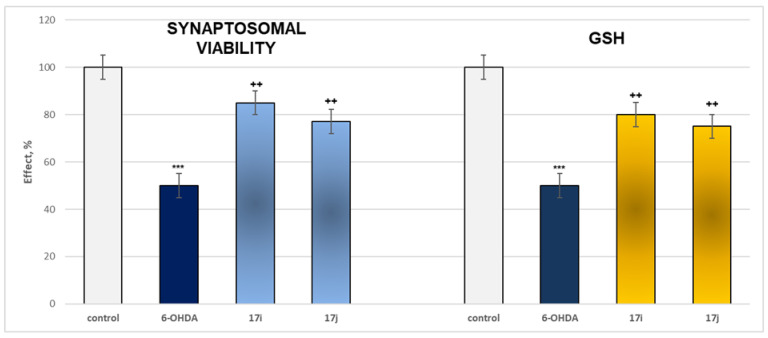
Effects of the most active representatives (at a concentration of 100 μM) in the model of 6-OHDA-induced neurotoxicity on parameters characterizing the functional–metabolic profile of synaptosomes, measured as synaptosomal viability and levels of reduced glutathione (GSH). *** *p* < 0.001 against the control (untreated synaptosomes); ++ *p* < 0.01 against the toxic agent (pure 6-OHDA).

The effects of the most active MAO B representatives and 6-OHDA applied alone on two evaluated parameters are presented in Figure 9.

The results indicate that when applied alone, 6-OHDA has a statistically significant neurotoxic effect by suppressing the synaptosomal viability and GSH levels by 50% when compared to the control (untreated synaptosomes) (Figure 9).

In isolated rat brain synaptosomes, under a model of 6-hydroxydopamine-induced neurotoxicity, the most active compounds exhibiting mild intrinsic neurotoxicity—**17i** and **17j**—demonstrated a pronounced and statistically significant neuroprotective effect. These agents preserved synaptosomal viability by 55–70% and maintained reduced glutathione (GSH) levels by 50–60% relative to the toxic control (pure 6-OHDA) (Figure 9).

- Neuroprotection properties in isolated rat brain mitochondria

When applied alone at a concentration of 75 µM, *t*-BuOOH exerts a pronounced and statistically significant neurotoxic effect, lowering GSH levels and increasing MDA production by 50% relative to the control (untreated mitochondria).

**Figure 10:**
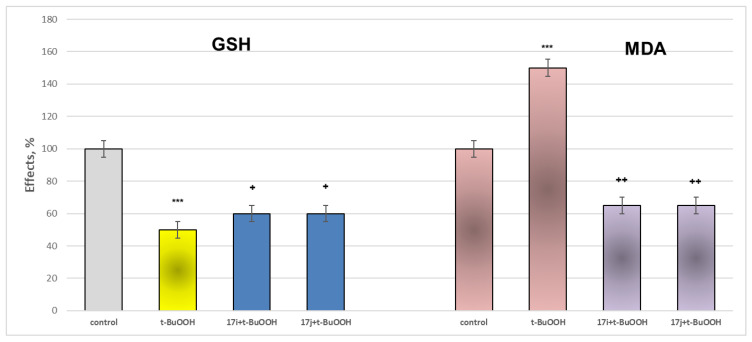
Effects of the most active representatives (at a concentration of 100 μM) in the model of t-BuOOH-induced neurotoxicity on parameters characterizing the functional–metabolic profile of mitochondria, measured as effects on levels of reduced glutathione (GSH) and production of MDA. *** *p* < 0.001 against the control (untreated mitochondria); + *p* < 0.05 and ++ *p* < 0.01 against t-BuOOH.

Within this oxidative stress model, the target derivatives exhibiting the lowest neurotoxicity and the most favorable MAO-B inhibitory activity, **17i** and **17j,** were evaluated. The results of the performed experiments are presented in Figure 10. Both compounds demonstrated a statistically significant neuroprotective effect.

The figure shows that **17i** and **17j** preserved GSH levels almost equally by 20% and reduced MDA production by 57% relative to the toxic agent (pure *t*-BuOOH) (Figure 10).

- Neuroprotection properties in isolated rat brain microsomes

**Figure 11:**
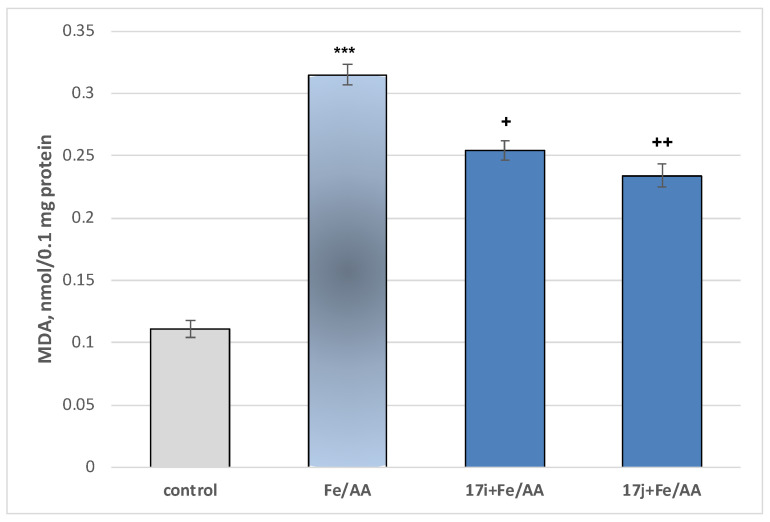
Effects of the most active representatives (at a concentration of 100 μM) in the model of Fe^2+^/AA-induced oxidative stress on parameters characterizing the functional–metabolic profile of microsomes, measured as effects on the production of MDA. *** *p* < 0.001 against the control (untreated microsomes); + *p* < 0.05 and ++ *p* < 0.01 against the toxic agent Fe^2+^/AA.

In our study, the experimental procedure showed that exposure of brain microsomes to the Fe^2+^/ascorbate system—conditions that promote continuous in situ •OH generation—resulted in a statistically significant elevation of malondialdehyde (MDA) levels by 184% relative to untreated controls (Figure 11).

When attempting to evaluate the neuroprotective properties of the newly synthesized derivatives, the latter were incubated at a concentration of 100 μM in combination with Fe^2+^/AA. The results of the performed experiments are presented in Figure 11.

Under the non-enzymatic iron/ascorbate-induced lipid peroxidation model, the compounds exhibiting the weakest pro-oxidant activity—**17i** and **17j**—demonstrated a measurable antioxidant effect relative to the iron/ascorbate control. Specifically, **17i** produced a statistically significant reduction in MDA levels of 19%, and **17j** of 26%, compared with the toxic agent (Figure 11).

The most active compounds, **17i** and **17j**, when applied individually at a concentration of 100 μM, exhibit low to moderate yet statistically significant neurotoxic and pro-oxidant effects in isolated rat brain synaptosomes, mitochondria, and microsomes.

### 2.3. In Silico Characterization

#### 2.3.1. In Silico Assessment of the Possible Toxicity

Both compounds were evaluated for potential toxicological liabilities using an in silico toxicity assessment performed with the Derek Nexus platform, version 2024.1, database version 5.0.1 (Lhasa Limited, Leeds, UK). This analysis screened each compound against a comprehensive panel of more than 55 toxicity endpoints. For both compounds, no toxicity alerts were identified for the endpoints listed in Table 3.

The corresponding alert image, available in the evaluated molecules together with its occurrence, is presented in Table 4.

The structural representation of the key moiety, considered as a reason for the predicted hepatotoxicity and carcinogenic properties, is visible in Table 5.

#### 2.3.2. In Silico Metabolic Profiling

The predicted outcomes generated by Meteor are illustrated in Figure S5 for **5i** and Figure S6 for **5j** in the Supplementary File.

## 3. Discussion

### 3.1. Chemistry

The synthesis of the target hydrazide follows the classic Paal–Knorr condensation of a dicarbonyl derivative and an amino functionality. As an amino partner for condensation, 5-aminopentanoic acid was applied. This molecule is a delta-amino acid comprising pentanoic acid with an amino substituent at C-5. The compound is considered a methylene homolog of gamma-aminobutyric acid (GABA) and is characterized by the appearance of weak GABA agonistic activity. In the human organism, it plays a role of a human metabolite.

A group of azomethines of the target hydrazide was additionally obtained, aiming to enrich the available moieties of such compounds in the search for a defined leader in the design of new, more potent, and safe pyrrole derivatives with improved neurological effects.

The applied synthetic protocols enabled the isolation of the target compounds in high purity, with yields ranging from 52 to 89%, indicating a generally efficient and reproducible approach. To further optimize the synthesis of the most promising derivatives, modifications, such as microwave-assisted heating, could be employed, potentially enhancing reaction kinetics and overall yields while minimizing energy consumption and reaction times.

### 3.2. Pharmacological Studies

#### 3.2.1. Evaluation of MAO Inhibitory Activity Through In Vitro Methodology

Neurodegenerative diseases, including Parkinson’s and Alzheimer’s disease, remain among the most significant challenges in modern medicine. The progressive loss of neuronal function in these disorders profoundly impacts patient quality of life, and conventional therapies often fail to provide adequate symptomatic relief or to halt disease progression. Treatment of Parkinsonism is frequently focused on compensating for dopamine deficiency; however, such strategies do not effectively slow disease progression. Dopaminergic agonist therapy is commonly associated with adverse effects, particularly dyskinesia, which substantially diminishes patients’ quality of life.

Considering this, the inhibition of MAO-B represents an established and clinically validated approach in Parkinson’s disease therapy, as it can reduce dopamine breakdown and limit the formation of neurotoxic metabolites. While dual MAO-A/MAO-B inhibition has been proposed as a broader neuroprotective strategy, the present study specifically focuses on MAO-B selective agents and their potential to contribute to symptomatic treatment and neuroprotection.

Regarding MAO-B activity, two new molecules, **17i** and **17j**, displayed statistically significant inhibition, reducing enzymatic activity by 50% and 60%, respectively, compared with the control (pure *h*MAO-B). These effects are comparable to those of Selegiline, which inhibited MAO-B activity by 55% (Figure 3), with **17j** exhibiting even greater MAO-B inhibitory potency than Selegiline. As a result of additional studies conducted, it was found that **5j** had a better *h*MAO-B selectivity index (>225) compared to **5i**. Thus, the current findings support the identification of **17i** and **17j** as promising MAO-B selective inhibitors with potential relevance for the management of Parkinson’s disease.

#### 3.2.2. Neurotoxicity and Neuroprotection of the Target Azomethines in Brain Subcellular Fractions

The assessment of neurotoxicity in isolated brain subcellular fractions provides mechanistic insights into the cellular targets of neuroprotective compounds. In this study, synaptosomes, mitochondria, and microsomes isolated from rat brain tissue were employed to evaluate the effects of the pyrrole-based derivatives **17i** and **17j**. This approach allows a multidimensional analysis of compound effects on neuronal terminals, mitochondrial bioenergetics, and microsomal lipid peroxidation.

Synaptosomes, which retain functional neurotransmitter uptake, release, and enzymatic activity, were exposed to 100 μM of the compounds. Both **17i** and **17j** exhibited weak, yet statistically significant, intrinsic neurotoxicity. Under a 6-hydroxydopamine (6-OHDA)-induced neurotoxicity model, the derivatives significantly preserved synaptosomal integrity relative to the toxic control. These findings suggest that while baseline toxicity is low, the compounds efficiently mitigate oxidative and catecholaminergic stress at the synaptic level [5,16].

Mitochondria are central mediators of neuronal oxidative stress and apoptosis. In the tert-butylhydroperoxide (*t*-BuOOH)-induced oxidative stress model, **17i** and **17j** significantly maintained mitochondrial reduced glutathione (GSH) levels and decreased malondialdehyde (MDA) formation, demonstrating robust antioxidant and neuroprotective effects. Preservation of mitochondrial redox homeostasis and prevention of lipid peroxidation highlight the compounds’ ability to counteract ROS-induced damage, a key driver of neurodegeneration [17,18].

Microsomes, enriched in endoplasmic reticulum membranes, are highly sensitive to non-enzymatic Fe^2+^/ascorbate-induced lipid peroxidation. Both derivatives significantly reduced MDA formation, indicating potent antioxidant activity at the level of membrane lipids. Protection of microsomal integrity suggests that the compounds may mitigate peroxidative damage associated with neurotransmitter metabolism and cellular signaling [16].

The observed neuroprotection is likely mediated via two complementary mechanisms: (i) direct scavenging of reactive oxygen species (ROS), including hydroxyl radicals generated in Fenton-type reactions, and (ii) preservation of intracellular GSH, the primary nucleophile responsible for ROS detoxification. These mechanisms align with their selective MAO-B inhibition, reducing the formation of toxic metabolites and ROS during 6-OHDA degradation [5].

Protection was most pronounced in mitochondria and microsomes, consistent with their central roles in ROS generation and oxidative stress propagation. Synaptosomal protection, while significant, was comparatively lower, reflecting the vulnerability of neurotransmitter-dependent terminals. Collectively, these findings suggest that **17i** and **17j** possess a favorable neuroprotective profile with low intrinsic toxicity, supporting their potential as lead compounds for the development of therapies targeting oxidative stress-driven neurodegenerative disorders, including Parkinson’s and Alzheimer’s diseases [16,17,18].

### 3.3. In Silico Toxicity Assessment and Metabolic Profiling

Analysis using the Derek knowledge base revealed that neither **17i** nor **17j** triggered any toxicity alerts across all evaluated endpoints, indicating the absence of structural features associated with recognized toxicological risks. Both compounds contain a hydrazine moiety, classified as a category A contact allergen, based on human and animal data [19]. Furthermore, the furan ring in **17i** is associated with hepatotoxicity and carcinogenicity, as furan undergoes metabolic activation in the liver, producing reactive intermediates that damage tissue; chronic exposure in rodents increases liver tumor incidence, leading to its IARC classification as possibly carcinogenic to humans (Group 2B) [20].

Metabolic predictions using Meteor Nexus suggested that the primary biotransformation for both **17i** and **17j** involves hydrolysis of the acyclic carboxylic ester at the third position of the pyrrole ring, with high probability scores (716 for **17i** and 641 for **17j**). This indicates ester hydrolysis as the dominant metabolic pathway, supporting potential in vivo biotransformation and elimination routes.

The collective results revealed that **17i** and **17j** possess a favorable neuroprotective profile with low intrinsic toxicity, supporting their potential as lead compounds for neurodegenerative disorders, like Parkinson’s and Alzheimer’s diseases [5,16,17,18,19,20].

## 4. Materials and Methods

### 4.1. Chemistry

The structures of the newly synthesized compounds were characterized using IR, ^1^H/^13^C NMR, and mass spectrometry. IR spectra (400–4000 cm^−1^) were recorded on a Nicolet iS10 FT-IR spectrophotometer (Thermo Fisher Scientific, Waltham, MA, USA) using ATR-FTIR adapter. NMR spectra were obtained on a 600 MHz Bruker-Spectrospin WM600 MHz spectrometer (Bruker Switzerland AG, Faelanden, Switzerland), with TMS applied as an internal standard. Mass spectra were recorded by an Agilent 6410 triple quadrupole mass spectrometer (LCMS) with an electrospray ionization (ESI) interface (Santa Clara, CA, USA). Reaction progress and compound purity were monitored by TLC on silica gel plates TLC-Cards, Silica gel 60 F_254_, 1.05554 (Merck, Darmstadt, Germany) using CHCl_3_/C_2_H_5_OH as the eluent. Melting points were determined in open capillaries. Chemical names follow IUPAC nomenclature generated by ChemBioDraw Ultra software, Version 11.0 (CambridgeSoft, Cambridge, MA, USA). All reagents were commercially obtained from Merck and used without further purification.

#### 4.1.1. Synthesis of the Initial N-Pyrrolyl Carboxylic Acid **16**

The initial N-pyrrolylcarboxylic acid (5-(5-(4-bromophenyl)-3-(ethoxycarbonyl)-2-methyl-1H-pyrrol-1-yl)pentanoic acid) was obtained by classical Paal–Knorr synthesis using 0.1 mol 1,4-diketone (ethyl 2-acetyl-4-(4-bromophenyl)-4-oxobutanoate) and 1.2 mol of 5-aminovaleric acid (5-AVA), which are dissolved in 30 mL of glacial acetic acid. The mixture was heated and stirred for 5 h. Subsequently, the mixture was cooled at room temperature and poured into ice water. The precipitate was washed with hexane and dried.

5-(5-(4-bromophenyl)-3-(ethoxycarbonyl)-2-methyl-1*H*-pyrrol-1-yl)pentanoic acid (**16**)

IR (cm^−1^): 3345 (OH), 1771 (C=O), 1688 (COOC_2_H_5_), 1244 (C-O), 834 (p-substituted C_6_H_4_), 547 (C-Br); ^1^H NMR (δH, 400 MHz, DMSOd_6_): 1.29 [t, 3H, CH_2_CH_3_], 1.52 [m, 2H, CH_2_CH_2_CH_2_CH_2_], 1.74 [m, 2H, CH_2_CH_2_CH_2_CH_2_], 2.30 [t, 2H, CH_2_-CO_2_H], 2.47 [s, 3H, CH_3_(2)], 4.16 [t, 2H, N–CH_2_], 4.30 [q, 2H, CH_2_CH_3_], 6.78 [s, 1H, H(4)], 7.66 [d, 2H, H(3′), H(5′)], 7.78 [d, 2H, H(2′), H(6′)], 11.00 [br s, 1H, OH]; ^13^C NMR (100 MHz, DMSOd_6_): δ 178.4, 165.9, 142.1, 132.7, 132.1 (2C), 132.0, 128.3 (2C), 123.1, 108.2, 104.7, 60.9, 48.1, 33.7, 31.4, 22.6, 14.1 and 11.8; LC-MS (ESI): [M+H]+ 408.29.

#### 4.1.2. Synthesis of the Target Hydrazide **17**

In the next step, the pyrrole-based acid (0.007 mol) is esterified by reacting with thionyl chloride and absolute ethanol to obtain the corresponding intermediate ester. Subsequently, the N-pyrrolylcarboxylic acid ethyl ester without purification (5-(5-(4-bromophenyl)-3-(ethoxycarbonyl)-2-methyl-1H-pyrrol-1-yl)pentanoic acid) reacted with an excess of hydrazine hydrate to give the new pyrrole hydrazide 17 (ethyl 5-(4-bromophenyl)-1-(5-hydrazinyl-5-oxopentyl)-2-methyl-1H-pyrrole-3-carboxylate) in 20 mL of absolute ethanol.

ethyl 5-(4-bromophenyl)-1-(5-hydrazinyl-5-oxopentyl)-2-methyl-1*H*-pyrrole-3-carboxylate (**17**)

IR (cm^−1^): 3367 (NH), 2982 (CH_3_ and CH_2_), 1684 (COOC_2_H_5_), 1666 (Amide I), 1643 (Amide II), 1252 (C-O), 1073 (C-N), 812 (p-substituted C_6_H_4_), 546 (C-Br); ^1^H NMR (δH, 400 MHz, DMSOd_6_): 1.29 [t, 3H, CH_2_CH_3_], 1.53 [m, 2H, CH_2_CH_2_CH_2_CH_2_], 1.74 [m, 2H, CH_2_CH_2_CH_2_CH_2_], 2.0 [br s, 2H, NH_2_], 2.34 [t, 2H, CH_2_–CONHNH_2_], 2.47 [s, 3H, CH_3_(2)], 4.16 [t, 2H, N–CH2], 4.30 [q, 2H, CH2CH3], 6.78 [s, 1H, H(4)], 7.66 [d, 2H, H(3′), H(5′)], 7.78 [d, 2H, H(2′), H(6′)], 8.0 [br s, 1H, CONH]; ^13^C NMR (100 MHz, DMSOd_6_): δ 176.6, 165.9, 142.1, 132.7, 132.1 (2C), 132.0, 128.3 (2C), 123.1, 108.2, 104.7, 60.9, 48.1, 37.9, 31.9, 23.8, 14.1 and 11.8; LC-MS (ESI): [M+H]+ 422.32.

#### 4.1.3. General Synthesis of the New Hydrazones **17a**–**17j**

In the present synthetic scheme, 10 new hydrazide–hydrazones were obtained. Their preparation was carried out by condensation of the hydrazide (**17**) with different aldehydes (**a**-**j**). In this type of reaction, a Schiff base (azomethine group) is formed. For each individual reaction, equimolar amounts of 0.2 mM of hydrazide and the corresponding carbonyl, as well as 2 mL of glacial acetic acid medium, were used.

ethyl 1-(5-(2-benzylidenehydrazinyl)-5-oxopentyl)-5-(4-bromophenyl)-2-methyl-1*H*-pyrrole-3-carboxylate (**17a**)

IR (cm^−1^): 3191 (NH), 2976 (CH_3_ and CH_2_), 1693 (COOC_2_H_5_), 1667 (Amide I), 1598 (Amide II), 1246 (C-O), 815 (p-substituted C_6_H_4_), 544 (C-Br); ^1^H NMR (δH, 400 MHz, DMSOd_6_): 1.29 [t, 3H, CH_2_CH_3_], 1.53 [m, 2H, CH_2_CH_2_CH_2_CH_2_], 1.74 [m, 2H, CH_2_CH_2_CH_2_CH_2_], 2.34 [t, 2H, CH_2_–CONH–], 2.47 [s, 3H, CH_3_(2)], 4.16 [t, 2H, N–CH_2_], 4.30 [q, 2H, CH_2_CH_3_], 6.78 [s, 1H, H(4)], 7.52 [d, 2H, H(3″), H(4″), H(5″)], 7.66 [d, 2H, H(3′), H(5′)], 7.78 [d, 2H, H(2′), H(6′)], 7.83 [d, 2H, H(2″), H(6″)], 8.0 [br s, 1H, CONH], 8.36 [s, 1H, CH=N]; ^13^C NMR (100 MHz, DMSOd_6_): δ 167.0, 165.9, 144.1, 142.1, 133.7, 132.7, 132.1 (2C), 132.0, 131.0, 129.2 (2C), 128.8 (2C), 128.3 (2C), 123.1, 108.2, 104.7, 60.9, 48.1, 38.2, 31.9, 23.8, 14.1 and 11.8; LC-MS (ESI): [M+H]+ 510.43.

ethyl 5-(4-bromophenyl)-1-(5-(2-(2-hydroxybenzylidene)hydrazinyl)-5-oxopentyl)-2-methyl-1*H*-pyrrole-3-carboxylate (**17b**)

IR (cm^−1^): 3424 (OH), 3279 (NH), 2975 (CH_3_ and CH_2_), 1693 (COOC_2_H_5_), 1620 (Amide I), 1571 (Amide II), 1249 (C-O), 832 (p-substituted C_6_H_4_), 526 (C-Br); ^1^H NMR (δH, 400 MHz, DMSOd_6_): 1.29 [t, 3H, OCH_2_CH_3_], 1.53 [m, 2H, CH_2_CH_2_CH_2_CH_2_], 1.74 [m, 2H, CH_2_CH_2_CH_2_CH_2_], 2.34 [t, 2H, CH_2_–CONH–], 2.47 [s, 3H, CH_3_(2)], 4.16 [t, 2H, N–CH_2_], 4.30 [q, 2H, CH_2_CH_3_], 5.35 [s, 1H, OH], 6.78 [s, 1H, H(4)], 7.02 [m, 1H, H(3″)], 7.08 [m, 1H, H(5″)], 7.52 [d, 1H, H(4″)], 7.66 [d, 1H, H(6″)], 7.66 [d, 2H, H(3′), H(5′)], 7.78 [d, 2H, H(2′), H(6′)], 8.0 [br s, 1H, CONH], 8.78 [s, 1H, CH=N]; ^13^C NMR (100 MHz, DMSOd_6_): δ 167.0, 165.9, 157.2, 146.0, 142.1, 132.7, 132.4, 132.1 (2C), 132.0, 128.3 (2C), 127.5, 123.0, 121.4, 118.5, 117.8, 108.2, 104.7, 60.9, 48.1, 38.2, 31.9, 23.8, 14.1 and 11.8; LC-MS (ESI): [M+H]+ 526.43

ethyl 5-(4-bromophenyl)-1-(5-(2-(2-fluorobenzylidene)hydrazinyl)-5-oxopentyl)-2-methyl-1*H*-pyrrole-3-carboxylate (**17c**)

IR (cm^−1^): 3201 (NH), 2977 (CH_3_ and CH_2_), 1697 (COOC_2_H_5_), 1668 (Amide I), 1566 (Amide II), 1224 (C-O), 814 (p-substituted C_6_H_4_), 519 (C-Br); ^1^H NMR (δH, 400 MHz, DMSOd_6_): 1.29 [t, 3H, CH_2_CH_3_], 1.53 [m, 2H, CH_2_CH_2_CH_2_CH_2_], 1.74 [m, 2H, CH_2_CH_2_CH_2_CH_2_], 2.34 [t, 2H, CH_2_–CONH–], 2.47 [s, 3H, CH_3_(2)], 4.16 [t, 2H, N–CH_2_], 4.30 [q, 2H, CH_2_CH_3_], 6.78 [s, 1H, H(4)], 7.29 [m, 1H, H(5″)], 7.36 [m, 1H, H(3″)], 7.50 [d, 1H, H(4″)], 7.66 [d, 2H, H(3′), H(5′)], 7.78 [d, 2H, H(2′), H(6′)], 7.81 [d, 1H, H(6″)], 8.0 [br s, 1H, CONH], 8.36 [s, 1H, CH=N]; ^13^C NMR (100 MHz, DMSOd_6_): δ 167.0, 165.9, 159.6, 143.3, 142.1, 132.7, 132.6, 132.1 (2C), 132.0, 130.8, 128.3 (2C), 124.4, 123.0, 118.2, 115.6, 108.2, 104.7, 60.9, 48.1, 38.2, 31.9, 23.8, 14.1 and 11.8; LC-MS (ESI): [M+H]+ 528.42

ethyl 5-(4-bromophenyl)-1-(5-(2-(3-fluorobenzylidene)hydrazinyl)-5-oxopentyl)-2-methyl-1*H*-pyrrole-3-carboxylate (**17d**)

IR (cm^−1^): 3208 (NH), 2979 (CH_3_ and CH_2_), 1669 (COOC_2_H_5_), 1625 (Amide I), 1582 (Amide II), 1247 (C-O), 827 (p-substituted C_6_H_4_), 516 (C-Br); ^1^H NMR (δH, 400 MHz, DMSOd_6_): 1.29 [t, 3H, CH_2_CH_3_], 1.53 [m, 2H, CH_2_CH_2_CH_2_CH_2_], 1.74 [m, 2H, CH_2_CH_2_CH_2_CH_2_], 2.34 [t, 2H, CH_2_–CONH–], 2.47 [s, 3H, CH_3_(2)], 4.16 [t, 2H, N–CH_2_], 4.30 [q, 2H, CH_2_CH_3_], 6.78 [s, 1H, H(4)], 7.36 [m, 1H, H(4″)], 7.60 [m, 1H, H(6″)], 7.63 [d, 1H, H(5″)], 7.66 [d, 2H, H(3′), H(5′)], 7.78 [d, 2H, H(2′), H(6′)], 7.80 [d, 1H, H(2″)], 8.0 [br s, 1H, CONH], 8.36 [s, 1H, CH=N]; ^13^C NMR (100 MHz, DMSOd_6_): δ 167.0, 165.9, 163.0, 146.8, 142.1, 135.3, 132.7, 132.1 (2C), 132.0, 130.4, 128.3 (2C), 124.8, 117.8, 114.0, 108.2, 104.7, 60.9, 48.1, 38.2, 31.9, 23.8, 14.1 and 11.8; LC-MS (ESI): [M+H]+ 528.42

ethyl 5-(4-bromophenyl)-1-(5-(2-(4-methoxybenzylidene)hydrazinyl)-5-oxopentyl)-2-methyl-1*H*-pyrrole-3-carboxylate (**17e**)

IR (cm^−1^): 3234 (NH), 2956 (CH_3_ and CH_2_), 1689 (COOC_2_H_5_), 1669 (Amide I), 1567 (Amide II), 1245 (C-O), 832 (p-substituted C_6_H_4_), 546 (C-Br); ^1^H NMR (δH, 400 MHz, DMSOd_6_): 1.29 [t, 3H, CH_2_CH_3_], 1.53 [m, 2H, CH_2_CH_2_CH_2_CH_2_], 1.74 [m, 2H, CH_2_CH_2_CH_2_CH_2_], 2.34 [t, 2H, CH_2_–CONH–], 2.47 [s, 3H, CH_3_(2)], 3.83 [s, 3H, CH_3_], 4.16 [t, 2H, N–CH_2_], 4.30 [q, 2H, CH_2_CH_3_], 6.78 [s, 1H, H(4)], 7.06 [m, 2H, H(3″), H(5″)], 7.66 [d, 2H, H(3′), H(5′)], 7.78 [d, 2H, H(2′), H(6′)], 7.84 [d, 2H, H(2″), H(6″)], 8.0 [br s, 1H, CONH], 8.36 [s, 1H, CH=N]; ^13^C NMR (100 MHz, DMSOd_6_): δ 167.0, 165.9, 162.9, 144.1, 142.1, 132.7, 132.1 (2C), 132.0, 130.2 (2C), 128.3 (2C), 126.0, 123.0, 114.4 (2C), 108.2, 104.7, 60.9, 55.8, 48.1, 38.2, 31.9, 23.8, 14.1 and 11.8; LC-MS (ESI): [M+H]+ 540.46.

ethyl 5-(4-bromophenyl)-1-(5-(2-(4-(dimethylamino)benzylidene)hydrazinyl)-5-oxopentyl)-2-methyl-1H-pyrrole-3-carboxylate (**17f**)

IR (cm^−1^): 3266 (NH), 2979 (CH_3_ and CH_2_), 1682 (COOC_2_H_5_), 1602 (Amide I), 1521 (Amide II), 1242 (C-O), 809 (p-substituted C_6_H_4_), 544 (C-Br); ^1^H NMR (δH, 400 MHz, DMSOd_6_): 1.29 [t, 3H, CH_2_CH_3_], 1.53 [m, 2H, CH_2_CH_2_CH_2_CH_2_], 1.74 [m, 2H, CH_2_CH_2_CH_2_CH_2_], 2.34 [t, 2H, CH_2_–CONH–], 2.47 [s, 3H, CH_3_(2)], 3.06 [t, 2H, (CH_3_)_2_–N–Ar], 4.16 [t, 2H, N–CH_2_], 4.30 [q, 2H, CH_2_CH_3_], 6.78 [s, 1H, H(4)], 6.81 [d, 2H, H(3″), H(5″)], 7.50 [d, 2H, H(2″), H(6″)], 7.66 [d, 2H, H(3′), H(5′)], 7.78 [d, 2H, H(2′), H(6′)], 8.0 [br s, 1H, CONH], 8.36 [s, 1H, CH=N]; ^13^C NMR (100 MHz, DMSOd_6_): δ 167.0, 165.9, 153.4, 144.1, 142.1, 132.7, 132.1 (2C), 132.0, 128.3 (4C), 123.0, 123.2, 111.9 (2C), 108.2, 104.7, 60.9, 48.1, 41.3 (2C), 38.2, 31.9, 23.8, 14.1 and 11.8; LC-MS (ESI): [M+H]+ 545.39.

ethyl 5-(4-bromophenyl)-1-(5-(2-(3-formylbenzylidene)hydrazinyl)-5-oxopentyl)-2-methyl-1H-pyrrole-3-carboxylate (**17g**)

IR (cm^−1^): 3198 (NH), 2931 (CH_3_ and CH_2_), 1695 (COOC_2_H_5_), 1601 (Amide I), 1523 (Amide II), 1247 (C-O), 813 (p-substituted C_6_H_4_), 532 (C-Br); ^1^H NMR (δH, 400 MHz, DMSOd_6_): 1.29 [t, 3H, CH_2_CH_3_], 1.53 [m, 2H, CH_2_CH_2_CH_2_CH_2_], 1.74 [m, 2H, CH_2_CH_2_CH_2_CH_2_], 2.34 [t, 2H, CH_2_–CONH–], 2.47 [s, 3H, CH_3_(2)], 4.16 [t, 2H, N–CH_2_], 4.30 [q, 2H, CH_2_CH_3_], 6.78 [s, 1H, H(4)], 7.66 [d, 2H, H(3′), H(5′)], 7.71 [d, 1H, H(5″)], 7.78 [d, 2H, H(2′), H(6′)], 8.0 [br s, 1H, CONH], 8.05, [d, 1H, H(4″)], 8.11 [d, 1H, H(6″)], 8.36 [s, 1H, CH=N], 8.38 [d, 1H, H(2″)], 9.88 [s, 1H, CHO]; ^13^C NMR (100 MHz, DMSOd_6_): δ 191.0, 167.0, 165.9, 146.8, 142.1, 137.0, 135.0, 134.2, 132.7, 132.1 (2C), 132.0, 129.3, 129.1 (2C), 128.3 (2C), 123.0, 108.2, 104.7, 60.9, 48.1, 38.2, 31.9, 23.8, 14.1 and 11.8; LC-MS (ESI): [M+H]+ 553.50

ethyl 5-(4-bromophenyl)-2-methyl-1-(5-(2-((5-nitrofuran-2-yl)methylene) hydrazinyl)-5-oxopentyl)-1H-pyrrole-3-carboxylate (**17h**)

IR (cm^−1^): 3198 (NH), 2931 (CH_3_ and CH_2_), 1695 (COOC_2_H_5_), 1601 (Amide I), 1523 (Amide II), 1247 (C-O), 813 (p-substituted C_6_H_4_), 532 (C-Br); ^1^H NMR (δH, 400 MHz, DMSOd_6_): 1.29 [t, 3H, CH_2_CH_3_], 1.53 [m, 2H, CH_2_CH_2_CH_2_CH_2_], 1.74 [m, 2H, CH_2_CH_2_CH_2_CH_2_], 2.34 [t, 2H, CH_2_–CONH–], 2.47 [s, 3H, CH_3_(2)], 4.16 [t, 2H, N–CH_2_], 4.30 [q, 2H, CH_2_CH_3_], 6.52 [d, 1H, H(4″)], 6.78 [s, 1H, H(4)], 6.93 [d, 1H, H(3″)], 7.0 [br s, 1H, CONH], 7.66 [d, 2H, H(3′), H(5′)], 7.75 [d, 1H, H(5″)], 7.78 [d, 2H, H(2′), H(6′)], 8.45 [s, 1H, CH=N]; ^13^C NMR (100 MHz, DMSOd_6_): δ 167.0, 165.9, 149.1, 144.4, 142.1, 137.3, 132.7, 132.1 (2C), 132.0, 128.3 (2C), 123.0, 118.9, 112.6, 108.2, 104.7, 60.9, 48.1, 38.2, 31.9, 23.8, 14.1 and 11.8; LC-MS (ESI): [M+H]+ 500.39

ethyl 5-(4-bromophenyl)-1-(5-(2-(furan-2-ylmethylene)hydrazinyl)-5-oxopentyl)-2-methyl-1H-pyrrole-3-carboxylate (**17i**)

IR (cm^−1^): 3198 (NH), 2931 (CH_3_ and CH_2_), 1695 (COOC_2_H_5_), 1601 (Amide I), 1523 (Amide II), 1247 (C-O), 813 (p-substituted C_6_H_4_), 532 (C-Br); ^1^H NMR (δH, 400 MHz, DMSOd_6_): 1.29 [t, 3H, CH_2_CH_3_], 1.53 [m, 2H, CH_2_CH_2_CH_2_CH_2_], 1.74 [m, 2H, CH_2_CH_2_CH_2_CH_2_], 2.34 [t, 2H, CH_2_–CONH–], 2.47 [s, 3H, CH_3_(2)], 4.16 [t, 2H, N–CH_2_], 4.30 [q, 2H, CH_2_CH_3_], 6.78 [s, 1H, H(4)], 7.0 [br s, 1H, CONH], 7.09 [d, 1H, H(3″)], 7.59 [d, 1H, H(4″)], 7.66 [d, 2H, H(3′), H(5′)], 7.78 [d, 2H, H(2′), H(6′)], 8.45 [s, 1H, CH=N]; ^13^C NMR (100 MHz, DMSOd_6_): δ 167.0, 165.9, 152.0, 151.8, 142.1, 134.6, 132.7, 132.1 (2C), 132.0, 128.3 (2C), 123.0, 114.4, 114.3, 108.2, 104.7, 60.9, 48.1, 38.2, 31.9, 23.8, 14.1 and 11.8; LC-MS (ESI): [M+H]+ 538.44

ethyl 5-(4-bromophenyl)-2-methyl-1-(5-oxo-5-(2-((3-oxoindolin-2-yl)methylene) hydrazinyl)pentyl)-1H-pyrrole-3-carboxylate (**17j**)

IR (cm^−1^): 3198 (NH), 2931 (CH_3_ and CH_2_), 1695 (COOC_2_H_5_), 1601 (Amide I), 1523 (Amide II), 1247 (C-O), 813 (p-substituted C_6_H_4_), 532 (C-Br); ^1^H NMR (δH, 400 MHz, DMSOd_6_): 1.29 [t, 3H, CH_2_CH_3_], 1.53 [m, 2H, CH_2_CH_2_CH_2_CH_2_], 1.74 [m, 2H, CH_2_CH_2_CH_2_CH_2_], 2.34 [t, 2H, CH_2_–CONH–], 2.47 [s, 3H, CH_3_(2)], 4.0 [br s, 1H, NH(1″)], 4.1 [d, 1H, H(2″)], 4.16 [t, 2H, N–CH_2_], 4.30 [q, 2H, CH_2_CH_3_], 6.78 [s, 1H, H(4)], 6.88 [d, 1H, H(6″)], 6.93 [d, 1H, H(8″)], 7.0 [br s, 1H, CONH], 7.42 [d, 1H, H(7″)], 7.50 [d, 1H, CH=N], 7.57 [d, 1H, H(5″)], 7.66 [d, 2H, H(3′), H(5′)], 7.78 [d, 2H, H(2′), H(6′)]; ^13^C NMR (100 MHz, DMSOd_6_): δ 196.5, 167.0, 165.9, 160.6, 154.7, 142.1, 133.9, 132.7, 132.1 (2C), 132.0, 129.6, 128.3 (2C), 123.0, 120.5, 117.0, 113.4, 108.2, 104.7, 70.2, 60.9, 48.1, 38.2, 31.9, 23.8, 14.1 and 11.8; LC-MS (ESI): [M+H]+ 551.44.

### 4.2. Pharmacological In Vitro Evaluations

#### 4.2.1. Determination of Human Recombinant MAO-A/B Enzyme Activity

The activity of recombinant human MAO-A/B was determined fluorimetrically. Tyramine hydrochloride was used as a substrate. The activity is determined by the detection of H_2_O_2_ production. This production is, in turn, reported by binding to horseradish peroxidase using N-acetyl-3,7-dihydroxyphenoxazine (AmplexRed, Thermo Fisher Scientific, Waltham, MA, USA) [21].

#### 4.2.2. Neurotoxicity and Neuroprotection Assessment

Neurotoxicity and neuroprotection studies were performed using 20 rats obtained from the National Breeding Centre of the Bulgarian Academy of Sciences and housed under standard conditions with free access to food and water; all procedures complied with Ordinance No. 15 (SG No. 17, 2006) and European regulations for experimental animal welfare and were approved by the Bulgarian Food Safety Agency (Permission No. 304, valid until 28 June 2026). Rat brain synaptosomes and mitochondria were isolated by subcellular fractionation on a Percoll gradient according to Taupin et al. [21]. Neurotoxicity was induced by incubating synaptosomes with 6-hydroxydopamine (6-OHDA, 150 µM, 1 h) following Stokes et al. [14], and synaptosomal viability was assessed by the MTT assay at 580 nm [22]. Reduced glutathione (GSH) levels in synaptosomes were determined as described by Robyt et al. [23]. In isolated mitochondria, malondialdehyde (MDA) production and GSH levels were measured spectrophotometrically at 532 and 412 nm, respectively, according to Shirani et al. [24], after induction of oxidative stress with tert-butyl hydroperoxide (t-BuOOH, 75 µM) [16]. Rat brain microsomes were isolated as previously reported [25], and lipid peroxidation was induced by Fe^2+^/ascorbate (20 µM FeSO_4_, 0.5 mM ascorbic acid) [26]. Microsomal MDA levels were quantified at 535 nm using a molar extinction coefficient of 1.56 × 10^5^ M^−1^ cm^−1^ [26].

### 4.3. In Silico Toxicity Assessment

#### 4.3.1. Software and Configuration

Toxicity assessments were performed using Derek Nexus (Knowledge Base v6.4.1; Nexus v2.7.2, Lhasa Limited, Leeds, UK), an expert system tailored for mechanistic toxicity evaluation. This software employs a rule-based framework that draws on structural alerts sourced from experimental toxicology data, the scientific literature, and regulatory guidelines. Metabolic predictions were carried out with Meteor Nexus (Lhasa Limited, Leeds, UK), a system designed for Phase I and II biotransformation analysis. Meteor Nexus integrates empirical findings, peer-reviewed publications, and proprietary metabolic rules to predict potential metabolites and their formation pathways.

#### 4.3.2. Compound Input and Preparation

The pyrrole-based lead compound, as detailed in Table 1, was entered into Derek Nexus using its canonical SMILES string. This notation was generated and validated with ChemAxon’s MarvinSketch (v22.15). To ensure reproducibility, the compound’s stereochemistry and tautomeric forms were explicitly defined prior to analysis.

#### 4.3.3. Endpoint Evaluation

All toxicity endpoints available in the Derek Prediction module were systematically evaluated, including carcinogenicity (such as IARC Group classifications), genotoxicity (mutagenicity and clastogenicity), organ toxicity (covering the liver, kidney, and nervous system), reproductive and developmental toxicity, sensitization (skin and respiratory), and other endpoints, such as phototoxicity and phospholipidosis. Predictions were generated for both bacterial systems, primarily for Ames test relevance, and mammals (rat and human) to assess species-specific toxicological profiles. Prediction confidence levels were categorized as probable (strong structural or mechanistic evidence, ≥70% likelihood), plausible (moderate evidence, 30–69% likelihood), equivocal (insufficient or conflicting evidence, <30%), or negative (no alerts), which were assigned when the compound lacked structural motifs associated with toxicity in the Derek knowledge base, such as phototoxic arylpropanone or polycyclic aromatic fragments.

#### 4.3.4. Metabolic Analysis

Metabolic pathways were visualized as hierarchical trees, annotating the parent compound and its derivatives with the corresponding reaction types, including hydroxylation and ester hydrolysis. Mechanistic rationales, such as the involvement of CYP3A4 in aromatic oxidation, were documented, and the literature citations supporting the predicted biotransformations were curated. Competing metabolic reactions, such as glucuronidation versus sulfation, were also identified and highlighted for further analysis.

### 4.4. Statistical Methods

The results obtained from *h*MAO-A/B activity were statistically processed using GraphPad Prism 5.0 software. The results of the experiments performed on isolated brain sub-fractions were statistically processed using the ‘MEDCALC’ program with the non-parametric Mann–Whitney method at significance levels of *p* < 0.05, *p* < 0.01, and *p* < 0.001.

## 5. Conclusions

Newly synthesized pyrrole-based derivatives **17i** and **17j** were obtained in good yields (52–89%) and demonstrated selective MAO-B inhibition, comparable or superior to Selegiline. Neurotoxicity assessment in isolated rat brain subcellular fractions revealed minimal intrinsic toxicity, while both compounds exerted significant neuroprotective and antioxidant effects in synaptosomes, mitochondria, and microsomes under 6-OHDA, *t*-BuOOH, and Fe^2+^/ascorbate-induced oxidative stress models. These effects are likely mediated by ROS scavenging and the preservation of reduced glutathione (GSH). In silico analyses indicated no major structural toxicity alerts, although the hydrazine group may act as a contact allergen, and the furan moiety in **17i** carries hepatotoxic and carcinogenic risk. Metabolic predictions suggest ester hydrolysis as the primary biotransformation pathway. Collectively, **17i** and **17j** represent promising lead compounds for the development of therapeutics targeting oxidative stress-related neurodegenerative disorders, such as Alzheimer’s and Parkinson’s diseases.

## Figures and Tables

**Table 1:** Purity elucidation data for the target derivatives, including melting points, TLC characteristics (Rf), MS data, and yields.

ID	m.p. °C	R_f_	MS Data [M+H]^+^ (*m/z*)	Yields %
**16**	90.3–92.1	0.65	408.29	86
**17**	97.3–102.2	0.73	422.32	83
**17a**	121.8–123.4	0.79	510.43	88
**17b**	122.2–125.7	0.71	526.43	84
**17c**	127.8–131.2	0.73	528.42	81
**17d**	126.4–130.3	0.53	528.42	80
**17e**	157.6–158.9	0.75	540.46	72
**17f**	138.7–140.2	0.62	545.39	52
**17g**	166.2–168.1	0.67	553.50	64
**17h**	99.2–103.3	0.50	500.39	74
**17i**	144.5–147.3	0.68	538.44	72
**17j**	95.4–96.3	0.30	551.44	78

**Table 2:** IC_50_ (EC_50_) of the most active representatives, **5i** and **5j**, together with the standard Selegiline and Clorgiline on hMAO-A/B.

Compounds	IC_50_ (EC_50_), (µM ± SD) *h*MAO-A	IC_50_ (EC_50_), (µM ± SD) *h*MAO-B	SI
**5i**	>100	0.712 ± 0.19	>140
**5j**	>100	0.444 ± 0.12	>225
**Selegiline**	-	0.318 ± 0.18	
**Clorgiline**	18.74 ± 0.096	-	

**Table 3:** Endpoints with no toxicity alerts detected at the specified reasoning threshold.

Endpoint	Toxicity Allert
5alpha-reductase inhibition	Mutagenicity in vivo
Adrenal gland toxicity	Nephrotoxicit
Anaphylaxis	Neurotoxicity
Androgen receptor modulation	Non-specific genotoxicity in vitro
Bladder disorders	Non-specific genotoxicity in vivo
Bladder urothelial hyperplasia	Occupational asthma
Blood in urine	Ocular toxicity
Bone marrow toxicity	Oestrogen receptor modulation
Bradycardia	Oestrogenicity
Cardiotoxicity	Peroxisome proliferation
Cerebral oedema	Phospholipidosis
Chloracne	Photo-induced chromosome damage
Cholinesterase inhibition	Photo-induced non-specific genotoxicity
Chromosome damage in vitro	Photo-induced non-specific genotoxicity
Chromosome damage in vivo	Photoallergenicity
Cumulative effect on white cell count and immunology	Photocarcinogenicity
Cyanide-type effects	Photomutagenicity in vitro
Developmental toxicity	Phototoxicity
Glucocorticoid receptor agonism	Pulmonary toxicity
HERG channel inhibition in vitro	Respiratory sensitisation
High acute toxicity	Skin irritation/corrosion
Irritation (of the eye)	Skin sensitisation HPC
Irritation (of the gastrointestinal tract)	Splenotoxicity
Irritation (of the respiratory tract)	Teratogenicity
Kidney disorders	Testicular toxicity
Kidney function-related toxicity	Thyroid toxicity
Lachrymation	Uncoupler of oxidative phosphorylation
Methaemoglobinaemia	Urolithiasis
Mitochondrial dysfunction	

**Table 4:** Alert image and its occurrence in **17i** and **17j** (highlighted in gray).

Alert Description	Match with Query Compounds
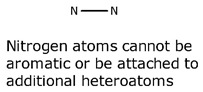	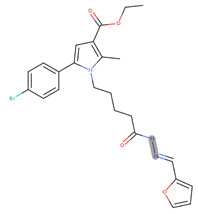
	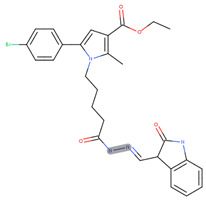

**Table 5:** Structural representation of the furan ring, a key moiety implicated in the hepatotoxic and carcinogenic properties of compound **17i**.

Alert Description	Match with 17i
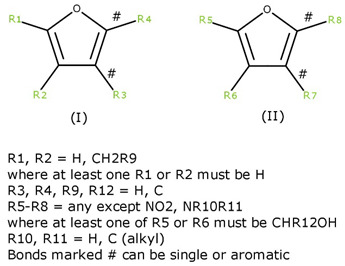	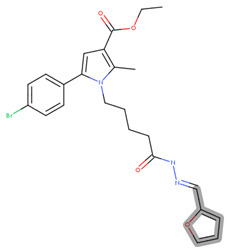

## Data Availability

The original contributions presented in this study are included in the article/Supplementary Material. Further inquiries can be directed to the corresponding author(s).

## Supplementary Materials

The following supporting information can be downloaded at: https://www.mdpi.com/article/10.3390/molecules31010186/s1, Figure S1: ^1^H-NMR of compound **17i**; Figure S2: ^13^C-NMR of compound **17i**; Figure S3: ^1^H-NMR of compound **17j**; Figure S4: ^13^C-NMR of compound **17j**; Figure S5: Metabolic profiles of compound **17i**; Figure S6: Metabolic profiles of compound **17j**.
