# Supplementary material for: Evaluation of Pyrrole Heterocyclic Derivatives as Selective MAO-B Inhibitors and Neuroprotectors
Source: Molecules. 2026 Jan 4;31(1):186. doi: 10.3390/molecules31010186 (PMC12787771; doi:10.3390/molecules31010186)
Supplement: Supplementary file 1 [file molecules-31-00186-s001.zip › molecules-4070979-supplementary.pdf]

Evaluation of pyrrole heterocyclic derivatives as selective MAO-B inhibitors and neuroprotectors

Maya Georgieva[1,*], Martin Sharkov [1], Emilio Mateev[1], Alexandrina Mateeva[1] and Magdalena Kondeva-Burdina[2]

[1] Department of Pharmaceutical chemistry, Faculty of Pharmacy, Medical University – Sofia, 2 Dunav str., 1000, Sofia, Bulgaria; mgeorgieva@pharmfac.mu-sofia.bg
[2] Department of Pharmacology, pharmacotherapy and toxicology, Faculty of Pharmacy, Medical University – Sofia, 2 Dunav str., 1000, Sofia, Bulgaria; mkondeva@pharmfac.mu-sofia.bg
* Correspondence: mgeorgieva@pharmfac.mu-sofia.bg

Table of content:

1. ^{1}H-NMR and ^{13}C-NMR spectra of compounds **17i** and **17j**
2. Metabolic profile of compound **17i**.
3. Metabolic profile of compound **17j**.

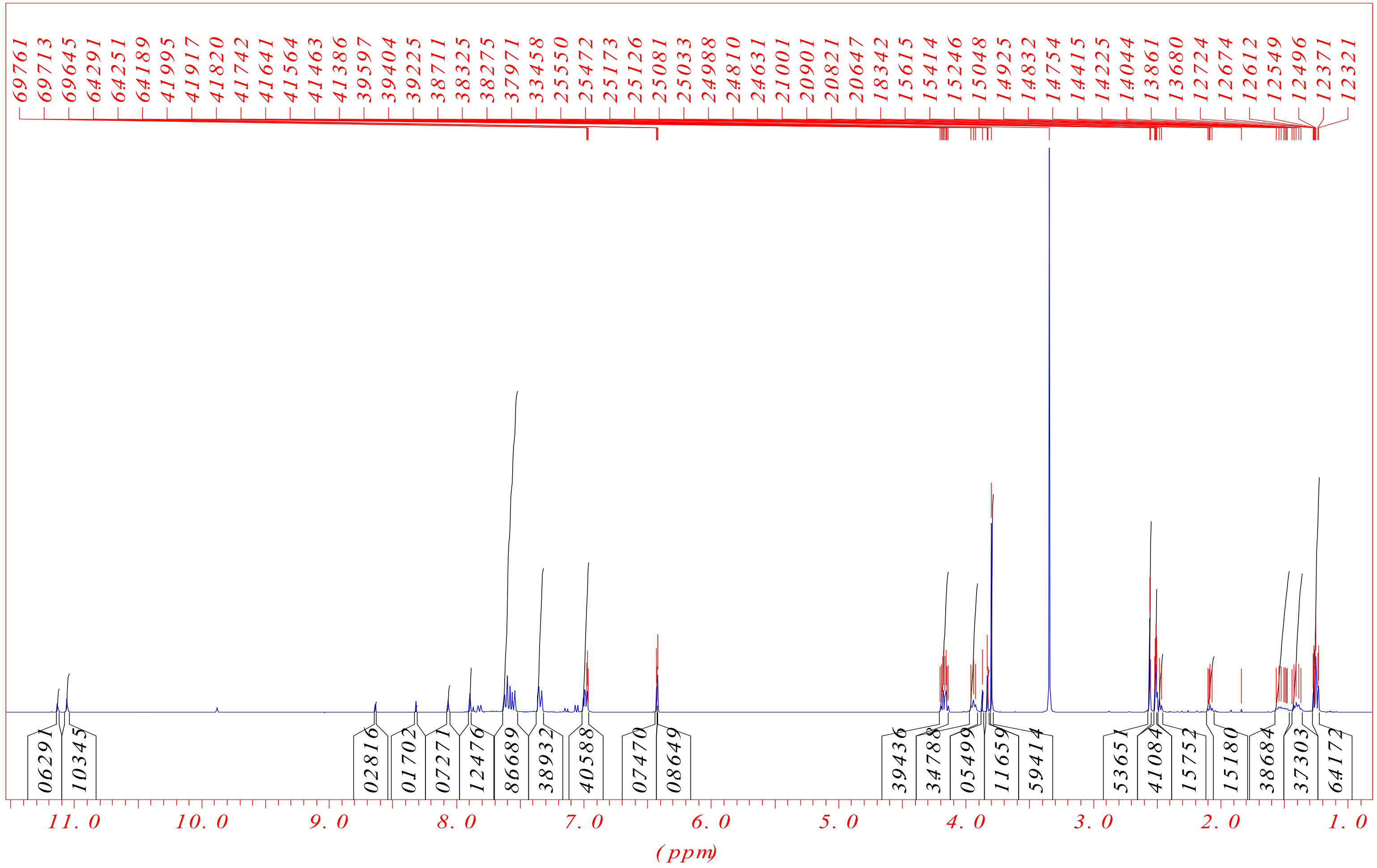

Supplementary Figure S1. ^{1}H-NMR of compound **17i**.

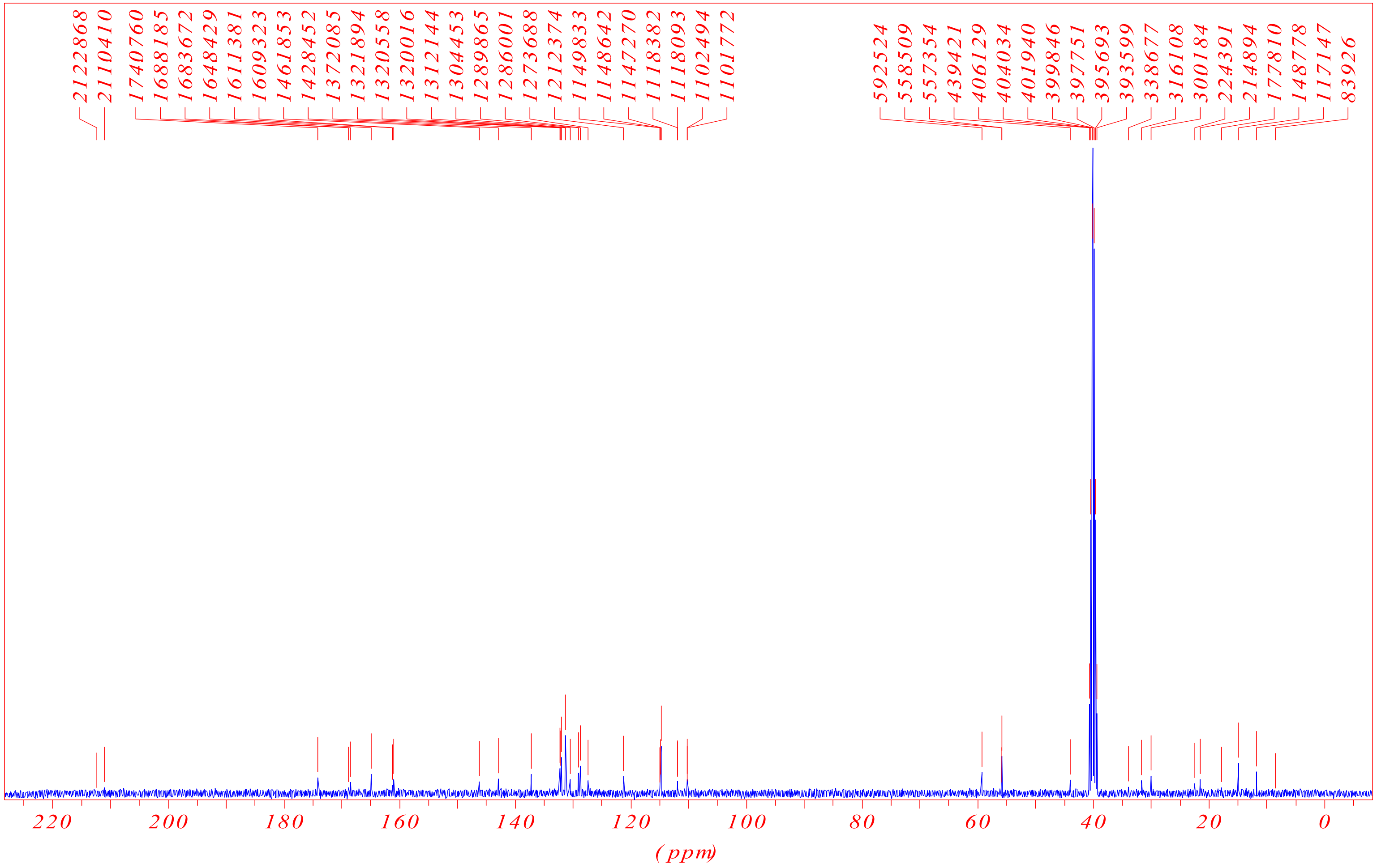

Supplementary Figure S2. ^{13}C-NMR of compound **17i**.

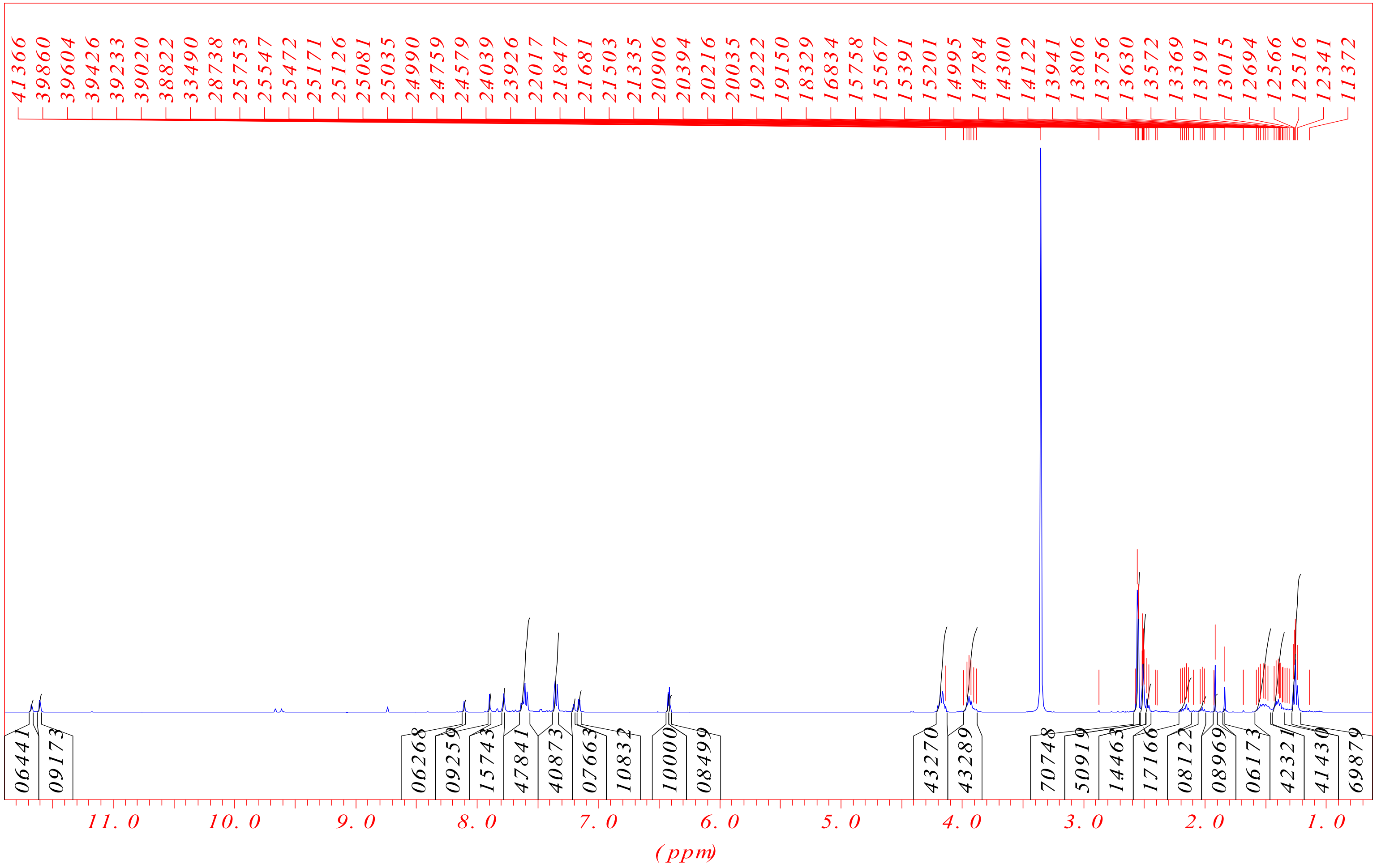

Supplementary Figure S3. ^{1}H-NMR of compound **17j**.

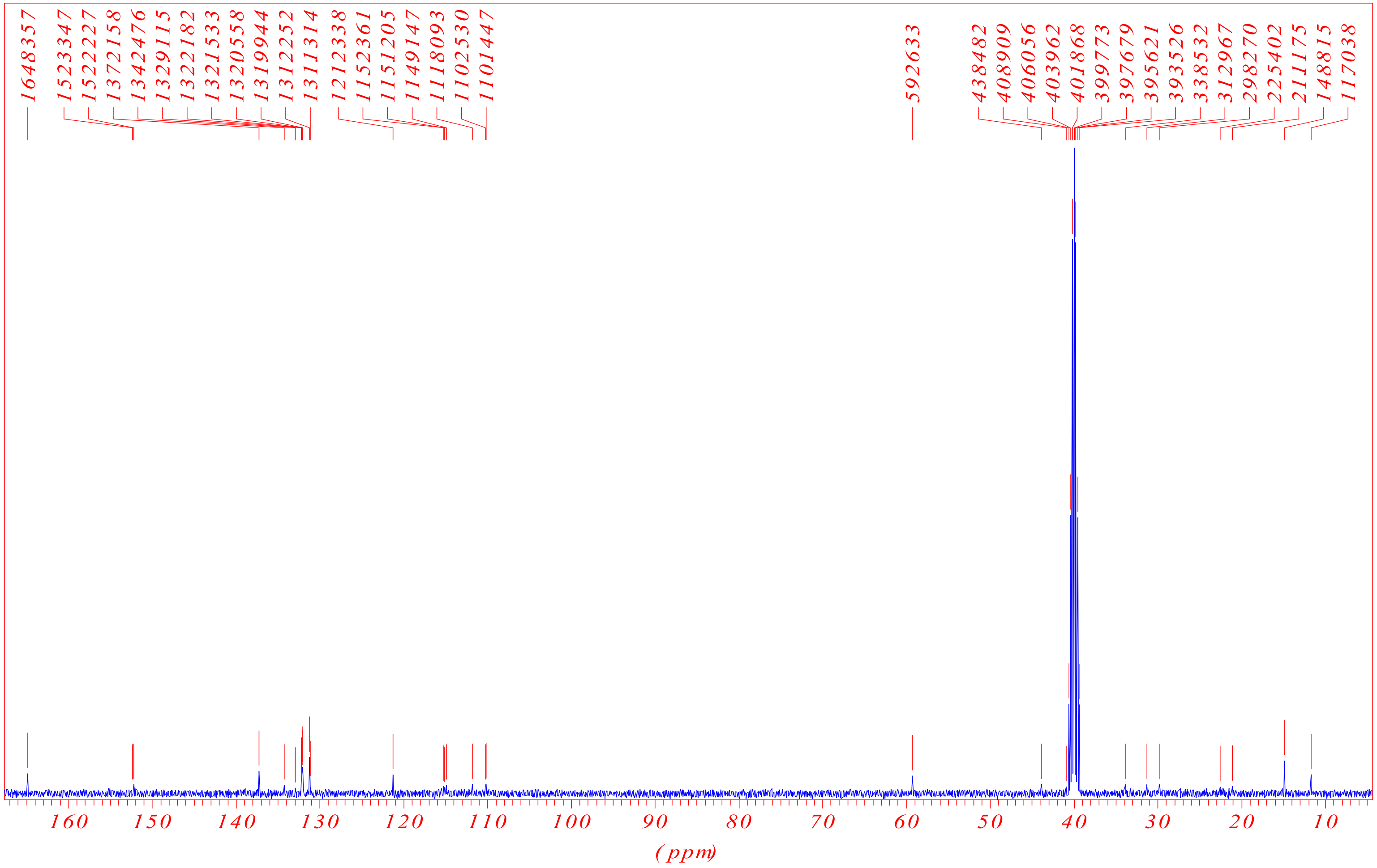

Supplementary Figure S4. ^{13}C-NMR of compound **17j**.

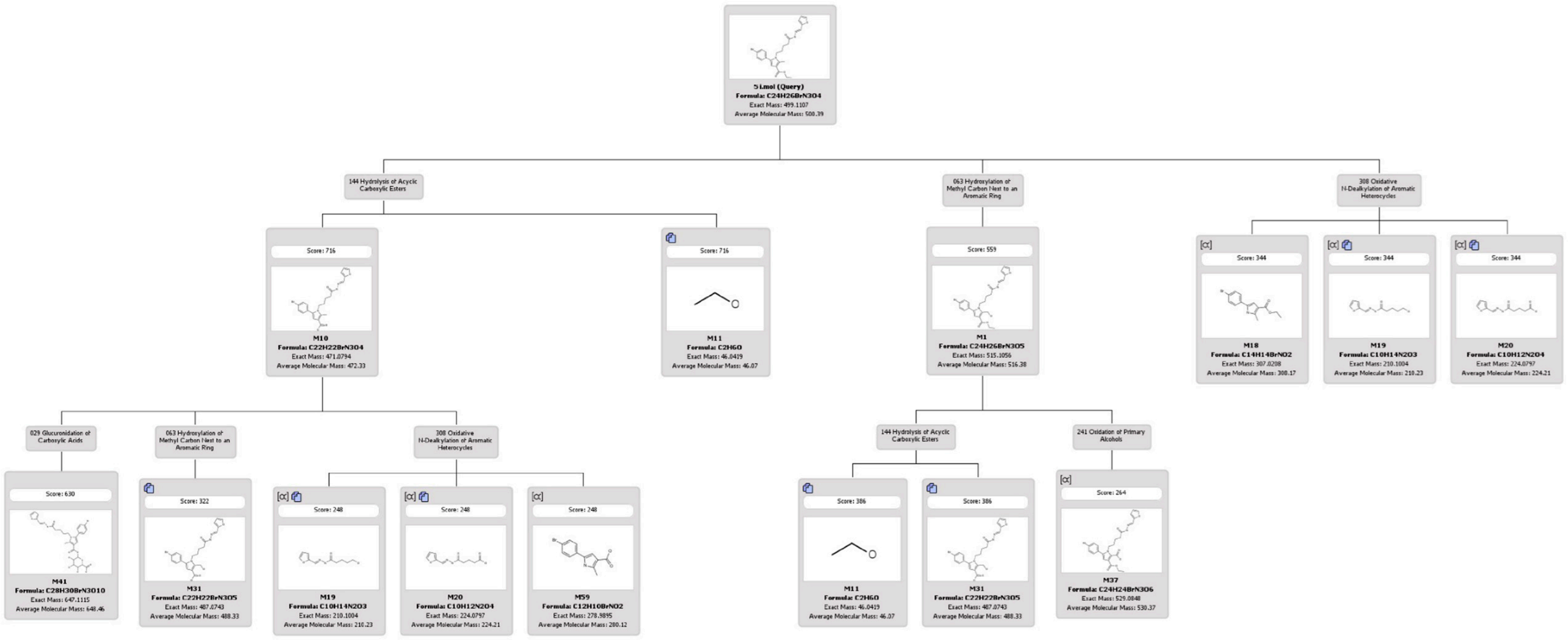

Supplementary Figure S5. Metabolic profiles of compound **17i**.

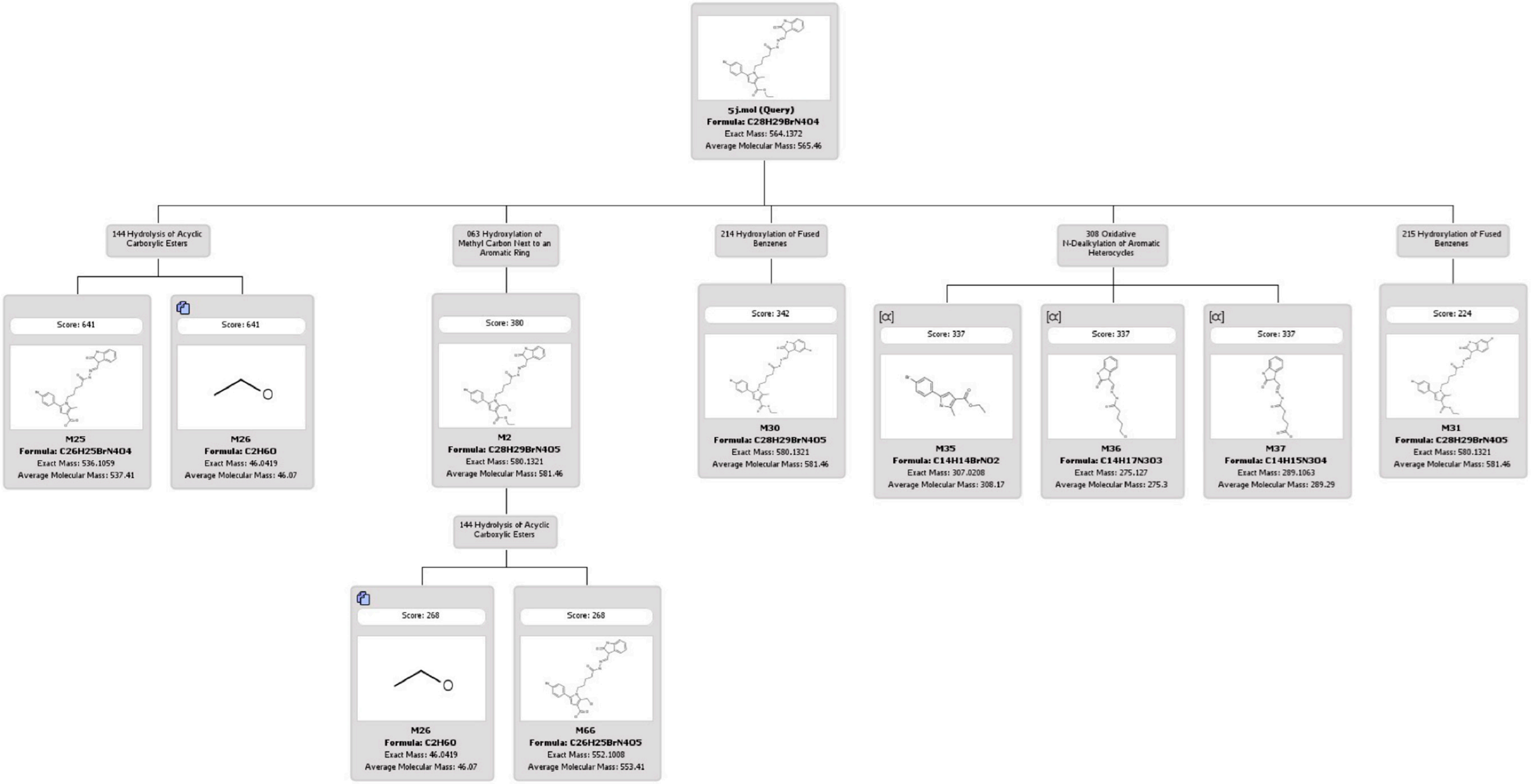

Supplementary Figure S6. Metabolic profiles of compound **17j**.
